# Evaluation of PBS Treatment and PEI Coating Effects on Surface Morphology and Cellular Response of 3D-Printed Alginate Scaffolds

**DOI:** 10.3390/jfb8040048

**Published:** 2017-11-01

**Authors:** María A. Mendoza García, Mohammad Izadifar, Xiongbiao Chen

**Affiliations:** 1 Departamento de Bioingeniería, Escuela de Ingeniería y Ciencias, Instituto Tecnológico y de Estudios Superiores de Monterrey Campus Estado de México, 52926 Cd López Mateos, Estado de México, Mexico; a01410878@itesm.mx; 2 Division of Biomedical Engineering, College of Engineering, University of Saskatchewan, Saskatoon, SK S7N5A9, Canada; mohammad.izadifar@usask.ca; 3 Department of Surgery, College of Medicine, University of Saskatchewan, Saskatoon, SK S7N5E5, Canada; 4 Department of Mechanical Engineering, College of Engineering, University of Saskatchewan, Saskatoon, SK S7N5A9, Canada

**Keywords:** alginate, tissue engineering, 3D printing, phosphate buffer saline, polyethyleneimine

## Abstract

Three-dimensional (3D) printing is an emerging technology for the fabrication of scaffolds to repair/replace damaged tissue/organs in tissue engineering. This paper presents our study on 3D printed alginate scaffolds treated with phosphate buffered saline (PBS) and polyethyleneimine (PEI) coating and their impacts on the surface morphology and cellular response of the printed scaffolds. In our study, sterile alginate was prepared by means of the freeze-drying method and then, used to prepare the hydrogel for 3D printing into calcium chloride, forming 3D scaffolds. Scaffolds were treated with PBS for a time period of two days and seven days, respectively, and PEI coating; then they were seeded with Schwann cells (RSC96) for the examination of cellular response (proliferation and differentiation). In addition, swelling and stiffness (Young’s modulus) of the treated scaffolds was evaluated, while their surface morphology was assessed using scanning electron microscopy (SEM). SEM images revealed significant changes in scaffold surface morphology due to degradation caused by the PBS treatment over time. Our cell proliferation assessment over seven days showed that a two-day PBS treatment could be more effective than seven-day PBS treatment for improving cell attachment and elongation. While PEI coating of alginate scaffolds seemed to contribute to cell growth, Schwann cells stayed round on the surface of alginate over the period of cell culture. In conclusion, PBS-treatment may offer the potential to induce surface physical cues due to degradation of alginate, which could improve cell attachment post cell-seeding of 3D-printed alginate scaffolds.

## 1. Introduction

One aim of tissue engineering is to create functional constructs of tissue or organs by combining cells with biomaterials for applications in regenerative medicine. The biomaterials act as templates to support and promote cell differentiation and proliferation for the regeneration of tissue. Currently, three-dimensional (3D) printing is an emerging technology that can help to achieve this aim [1]. This technique allows for the deposition of biomaterials and cells in a layer-by-layer manner, thus creating complex and functional constructs or scaffolds with good controllability and reproducibility [2,3].

In the development of tissue scaffolds, biocompability, biodegradability, mechanical properties, and scaffold structure are key factors of consideration [4]. Scaffolds must be biocompatible with the host tissue, to support/maintain cell functions, including adhesion and migration through the scaffold [5]. As scaffolds are used as temporary supports, they must be biodegradable and meanwhile allow cells to produce their own extracellular matrix (ECM) [4]. The scaffolds should also possess similar mechanical properties to the host tissue to provide mechanical support for cell growth and tissue regeneration, eventually restoring the functionality of the damaged tissue [6]. From a structural design perspective, the key factors are: interconnected pores, porosity and pore size of the scaffold, which can affect cell growth, morphology and proliferation in the scaffold [5]. As such, biomaterials should be non-immunogenic and biodegradable and able to mimic ECM to improve the scaffold interaction with the biological systems in the host tissue.

Recent studies in 3D printing offer an excellent opportunity to address some of the challenges faced by tissue engineering approaches [7]. Hydrogels made from alginate, have been widely used for 3D bioprinting. As such, these studies are diving into the benefits and disadvantages of the hydrogel, including strategies to improve its structural and degradation characteristics [8]. Approaches in different mechanical properties such as the influence of flow behavior of alginate-cell suspensions and its impact on cell viability and proliferation [9], and cell-laden tissue constructs with controllable degradation caused by an incubation of the (collagen/gelatin/alginate) hydrogel in a medium with sodium citrate [10], focus on mechanical properties which affect cell proliferation and the expression of biomolecules that may help improve the use of this hydrogel. Breakthroughs in applications such as drug screening, as well as regenerative medicine had been made. Some of these advances include cardiac alginate implants, in which bioprinting patterns on electrical/mechanical behavior was assessed [11], nano-reinforced hybrid cardiac patch was 3D bioprinted [12], and the preparation and characterization of alginate microspheres, where a controlled release and preserved biological activity was achieved [13]. All these advances in applications and structural properties are made taking into consideration the need for a suitable hydrogel which can create a scaffold that allows an organized regeneration, offering support as well as a suitable transport of nutrients, instead of trapping the cells and reducing their capacity to differentiate [14,15].

One of the applications of hydrogel scaffolds is peripheral nerve tissue engineering. Engineered hydrogel nerve scaffolds have shown potential to deliver living cells such as Schwann cells and to support the regrowth of axons for peripheral nerve tissue engineering [15]. Among different types of cells which are involved in nerve regeneration [16,17], Schwann cells, in response to nerve injury, undergo proliferation and phenotypical changes to prepare the local environment for axonal regeneration, which is considered as a critical process in nerve regeneration [14]. Studies have demonstrated that Schwann cells seeded on engineered nerve conduits could allow for bridging nerve defects and enhancing nerve regeneration [18,19]. Thus, Schwann cell response (e.g., attachment, elongation, proliferation) to a hydrogel scaffold is considered an important factor for the hydrogel biological performance assessment of a hydrogel peripheral nerve scaffold.

Alginate is an anionic polymer that has been widely used to create scaffolds due to its biocompatibility, ease of gelation, and low toxicity [20,21,22,23,24]. Alginate is typically used in the form of hydrogel, which is a cross-linked network of polymers with high water content [25]. Alginate has a quick gelation when exposed to divalent cations (through ionic crosslinking) such as calcium chloride, which allows for the establishment of gel matrix [26]. However, low mechanical properties and rapid degradation of ionically cross-linked alginate gels are issues for the fabrication of 3D scaffolds from alginate. For the improvement of this condition, the addition of polyethyleneimine (PEI) to alginate has been recently reported [23,27]. PEI is a synthetic polymer, which is weakly basic, aliphatic, nontoxic and polycationic [28]. Addition of PEI to alginate causes strong ionic interactions that result in a stable polymer, as the alginate neutralizes the positive charges of PEI [27,29].

Phosphate buffered saline (PBS), is a buffer solution which has similar osmolality and ion concentration to the human body. Ionically cross-linked alginate gels can be dissolved in physiological conditions, due to the release of divalent ions, caused by calcium ion exchange with monovalent cations such as sodium ions [24]. However, the effects of PBS and PEI coating on the 3D-printed scaffolds have not been documented in the literature. As inspired, we performed a study on the 3D printed alginate scaffolds treated with PBS and PEI coating and investigated into the effects of the treatment on the surface morphology and cellular response of the printed scaffolds. We hypothesize that PBS treatment may promote degradation of alginate, inducing physical surface cues, which may improve post cell seeding attachment of 3D-printed alginate scaffolds.

## 2. Materials and Methods

Medium viscosity alginate (sodium salt from brown algae) and calcium chloride were purchased from Sigma-Aldrich (St. Louis, MO, USA). PEI, M.W. 60,000, 50% *w*/*w* aq. soln. (Alfa Aesar, Haverhill, MA, USA), PBS (0.0067 M without calcium and magnesium at pH 7.0–7.2) (GE Healthcare Life Sciences, Mississauga, ON, Canada) and neuronal Schwann cell (RSC96) were used for experiments. High glucose DMEM (Gibco Thermo Fisher Scientific, Burlington, ON, Canada) with 10% fetal bovine serum (Gibco Thermo Fisher Scientific, Burlington, ON, Canada), and antibiotics (penicillin/streptomycin) (Gibco Thermo Fisher Scientific, Burlington, ON, Canada), were used for cell culture.

### 2.1. Preparation of Alginate and Its Solutions for 3D Printing

Medium viscosity sodium alginate was sterilized by using a freeze-drying method. Specifically, the alginate solution was prepared in deionized water at a concentration of 0.5% (*w*/*v*) and filtered by using sterile vacuum filters with a pore size of 0.2 μm; then the alginate solution was frozen in falcon tubes (previously weighed) at −40 °C for 3 days, followed by freeze-drying (−46 °C, <1 mbar) for 2 days. Eventually, the freeze-dried alginate was dissolved in sterile water to obtain a concentration of 2% (*w*/*v*) alginate solution for 3D printing.

Calcium chloride and PEI solutions were prepared at concentrations of 50 mM and 0.5%, respectively, followed by sterilization of the solution (filtration), using vacuum filters with pore size of 0.2 μm. Calcium chloride was used as the cross linker during 3D printing of scaffolds. Before 3D-printing, 12-well culture plates were coated with PEI over night to improve the attachment of the alginate filaments to the culture plate during printing, as per our previous study [27]. The idea behind this is that alginate has a negatively charged surface [1] and that PEI is positively charged, thus able to promote the attachment of alginate to the culture PEI coated plate when printing scaffolds [27].

### 2.2. Design and Fabrication of Scaffolds

**Figure 1:**
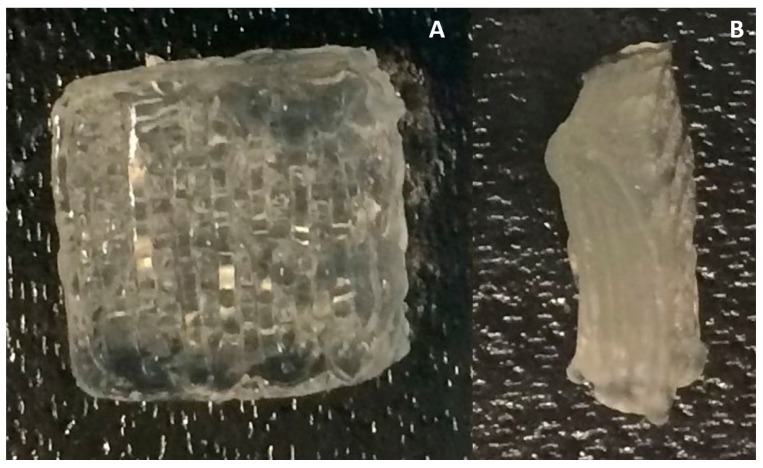
Three-dimensional-printed alginate scaffold of 14 layers (2.24 mm in height): (**A**) Top view and (**B**) side view.

In the design of scaffolds, the software of the 3D Bioplotter (EnvisionTEC, Gladbeck, Germany) was used to define the overall size of scaffolds as 8 mm (width) × 8 mm (length) and layer height as 4 mm. Also, the intra-strand space was defined as 1 mm and the angles of the two adjacent layers as 0°/90°. For the scaffold fabrication, the alginate solution, as prepared previously, was loaded to the cartridge of the 3D bioplotter and printed via a conical needle of 0.2 mm in diameter. The alginate solution was printed into the 12-well culture plates coated with PEI, as descripted previously, each of the plates containing the crosslinking solution of 50 mM calcium chloride with a volume of 1.1 mL. 3D-printing was performed under the following conditions: applied pressure of 0.1 bar, needle movement speed of 14 mm/s, needle offset from the plates of 0.06 mm, and room temperature. Scaffolds of 14 layers were printed for cell seeding, mechanical and swelling analysis. Two layer scaffolds were used for cell seeding analysis, for a better visualization of cell attachment and morphology. An example of a 14-layer scaffold is shown in Figure 1.

### 2.3. PBS and PEI Treatments of Scafolds

The printed scaffolds were treated with PBS over a time period of 0, 2 and 7 days, respectively. The treated scaffolds were then incubated at physiologically conditions of 37 °C and 5% CO_2_, then the solutions were removed and the scaffolds were frozen for 3 days, followed by freeze-drying for 2 days. After that, cell seeding was performed (see Section 2.5) and monitored using bright field microscope imaging over 7 days post cell-seeding.

For the PEI treatment, the PBS-treated scaffolds, as described above, were coated with PEI by adding 1 mL of 0.1% PEI directly to the scaffolds and incubated for 2 days. After that, the PEI was removed and then the scaffolds were freeze-dried similar to the ones described above. Then, cell seeding was performed on the scaffolds.

### 2.4. Surface Morphology Assessment

For surface morphology assessment, the PBS-treated scaffolds were gold-coated (Q150T, Quorum Technologies, Lewes, UK) and then imaged by scanning electron microscopy (SEM) (Hitachi SU8000, Tokyo, Japan) at 5 kV.

### 2.5. Cell Seeding

RSC96 neuronal Schwann cells were cultured in complete the Dulbecco's modified Eagle’s medium (DMEM), which contains 10% FBS and 1% antibiotics. At cell confluence of 80%, cells were trypsinized, and then suspended in the 10 mL of DMEM for cell seeding. Cell population density was adjusted before cell seeding. For this, 10 μL of the cell suspension was used for cell counting using hemocytometer and the number of cells was calculated as

(1) $$\frac{cells}{mL}=\frac{A+B+C+D}{4}\times 10^{4}\times dilution\;factor,$$

(2) $$dilution\;factor=\frac{total\;volume\;for\;cell\;counting}{cell\;suspension\;volume\;added},$$

where *A*, *B*, *C* and *D* are the quadrants of the hematocytometer, where cells are counted. Then, the suspension was centrifuged for 5 min at a speed of 800 rpm. The supernatant was removed, and the cell pellet was resuspended in the DMEM medium. The final cell population density was calculated by

(3) $$C_{1}V_{1}{=C}_{2}V_{2},$$

where *C*_1_ is total concentration of cell suspension, *V*_1_ is the volume of DMEM used for the resuspension of the cell pellet, *C*_2_ is concentration of cells for each scaffold and *V*_2_ is the volume of cell suspension for each scaffold. The volume of cell suspension (*V*_2_) was added to each scaffold and incubated for 2 h. Then, 1 mL of DMEM was added to each scaffold. Cell morphology and attachment were monitored for 7 days using bright field microscopy imaging. A cell density of 2 × 10^6^ cells/mL was used for the PBS treatment and 1 × 10^6^ cells/mL for the PEI treatment.

### 2.6. Swelling Analysis

The swelling performance printed alginate scaffolds was analyzed based on the measurements of mass changes during swelling over time. Scaffolds were crosslinked with 50 mM CaCl_2_ for 30 min after printing, and the initial weight of the dried scaffolds was obtained. Then, they were incubated in PBS (37 °C, 5% CO_2_) for 3 days. The swelling degree was calculated with the equation below:

(4) $$Swelling\;degree=\frac{{(W}_{0}-W_{i})}{W_{0}}\times 100\%,$$

where *W*_0_ is the initial weight of the scaffold before incubation in PBS, and *W_i_* is the weight of the scaffold during the incubation in PBS at different time points.

### 2.7. Mechanical Analysis

Mechanical stiffness, in terms of compressive modulus, of the PBS-treated scaffolds were measured by a Biodynamic^®^ Test Instrument (Bose, Eden Prairie, MN, USA). The top and side views of the scaffolds were imaged to determine the area and thickness (critical factors for calculating stress-strain curve) of the samples using the ImageJ software. Each scaffold was loaded in the machine, and was compressed by 1 mm (half of the height of the scaffolds for each measurement). Based on the force and displacement data recorded, the stress-strain curve was obtained from the results calculated by

(5) $$\sigma =\frac{F}{A}$$

(6) $$ɛ=\frac{\Delta_{l}}{L_{0}}$$

where *σ* is the stress, *ɛ* is the strain, *F* is the force applied to the scaffolds, *A* is the cross-section area of scaffolds and calculated in ImageJ (mm^2^), ∆*_l_* is the change of thickness of scaffolds and *L*_0_ is the initial thickness of scaffolds. From the obtained stress-strain curve, the slope of the linear part of the curve was calculated for the Young’s modulus of the scaffolds examined.

## 3. Results

### 3.1. Surface Morphology of Scaffolds by SEM Imaging

**Figure 2:**
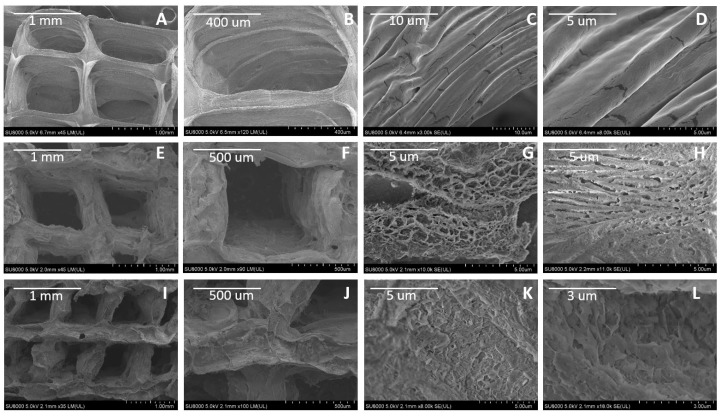
SEM images for surface analysis of the treated scaffolds: (**A**–**D**) non-treated scaffolds scale bars: (**A**) 1 mm; (**B**) 400 μm; (**C**) 10 μm and (**D**) 5 μm. (**E**–**H**) shows two days of PBS treatment at 37 °C, 5% CO_2_, with scales of: (**E**) 1 mm, (**F**) 500 μm and (**G**,**H**) 5 μm. (**I**–**L)** shows seven days PBS treated scaffolds, with scales bar of: (**I**) 1 mm; (**J**) 500 μm; (**K**) 5 μm and (**L**) 3 μm.

Figure 2 depicts the surface morphology of the control samples (Figure 2A–D) and PBS-treated scaffolds after 2 (Figure 2E–H) and 7 days (Figure 2I–L) of PBS treatment. As shown in Figure 2A–D, untreated alginate scaffolds present smooth surface with nearly no significant porosity. In contrast, the two-day PBS treatment results in a significant porous microstructure in the scaffold due to degradation (Figure 2E–H). Seven-day PBS treatment causes further degradation that leads to erosion-like structure of the alginate microstructure surface (Figure 2K,L) compared to those of two-day PBS treatment.

### 3.2. Cell Morpholoyg and Attachemnt

**Figure 3:**
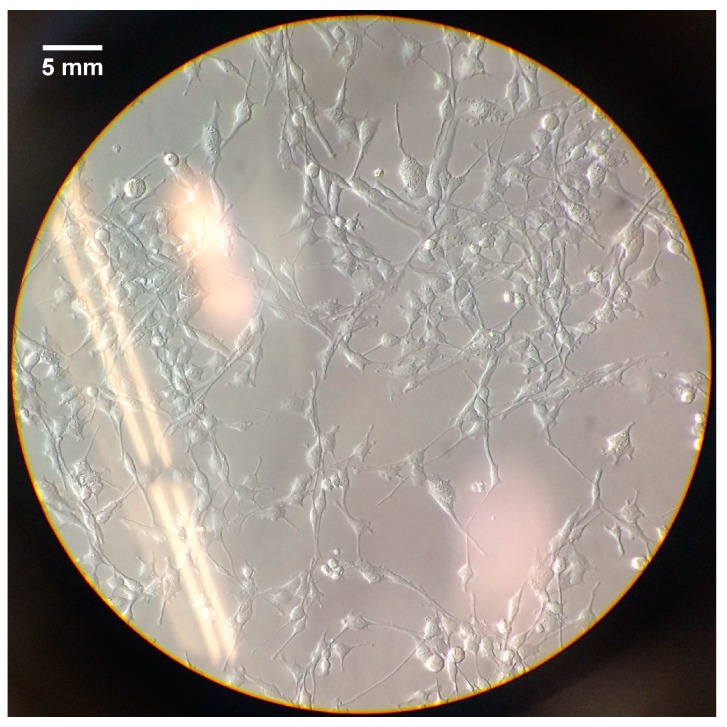
Two-dimensional/monolayer cell culture of Schwann cells.

**Figure 4:**
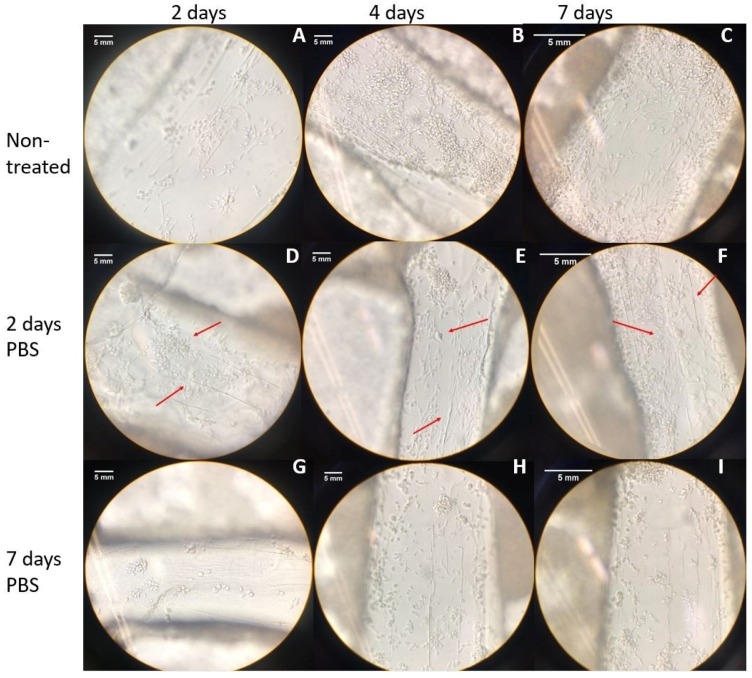
Cell morphology and attachment in scaffolds for untreated (**A**–**C**), two days PBS treatment (**D**–**F**) and seven days PBS treatment (**G**–**I**). All images are observed at 40× magnification. Red arrows indicate the cells, in place with a fibrous structure (scale bars = 5 mm).

**Figure 5:**
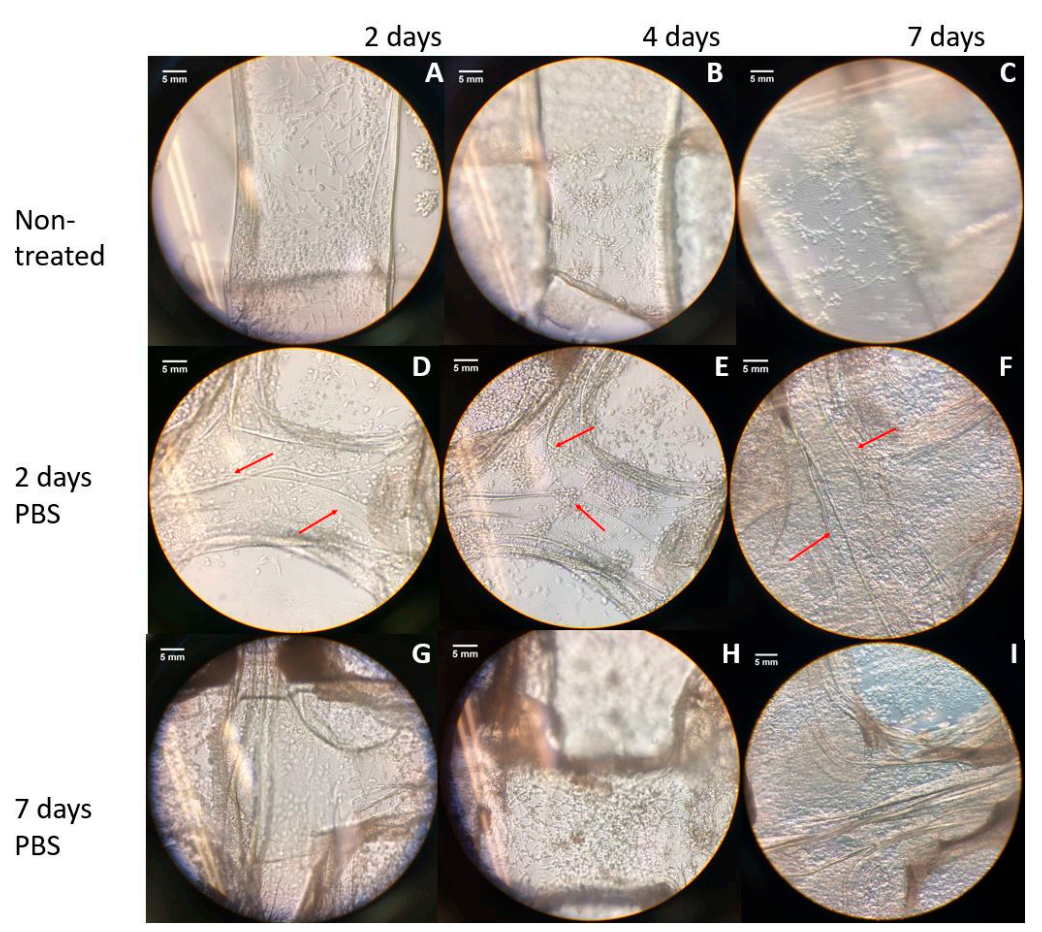
Cell morphology and attachment in scaffolds for the control samples (**A**–**C**), polyethyleneimine (PEI)-coated scaffolds with two-day PBS treatment (**D**–**F**), and PEI-coated scaffolds with seven-day PBS treatment (**G**–**I**). All images are observed through an inverted microscope with 40×. Red arrows are indicating the cells, in place with a fibrous structure (scale bars = 5 mm).

In this section, cell morphology and attachment will be shown for all the provided treatments. A control (Figure 3), is added in order to have a base of comparison for the morphology of the Schwann cells. In Figure 4 it is observed the results from the treatment of PBS and cell seeding, and in Figure 5 can be seen the outcome of the PEI coating treatment and posterior PBS treatment.

Figure 3 shows a 2D monolayer culture of Schwann cells (RSC96), in which the morphology of this cell line can be observed. This will act as a control for the cell morphology contemplated on all the hydrogel scaffolds.

In order to analyze the effects of the treatment with PBS on cell morphology and attachment, cell seeding was performed on the treated scaffolds and monitored for seven days. As it can be seen in Figure 4, cell attachment and growth on the cell-seeded scaffolds shows the improvement with time, which is particularly true in the two-day PBS treated scaffolds.

Figure 5 depicts the bright field microscope imaging of cells, indicating attachment and morphology of the cells in PEI-coated scaffolds, which were then treated with PBS for 2 and 7 days, compared to control samples over 7 days post cell seeding.

### 3.3. Mechanical and Swelling Analysis

**Figure 6:**
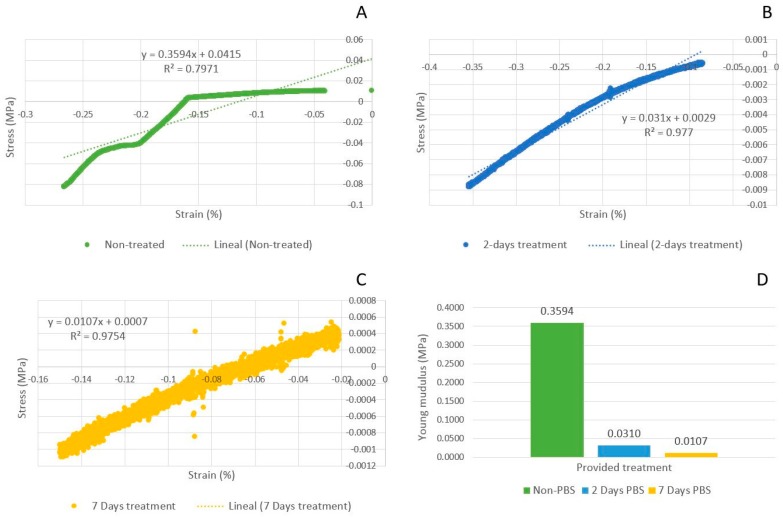
Young’s modulus of the 2% alginate scaffolds, crosslinked in 50 mM CaCl_2_. (**A**) Non-treated scaffold modulus is 0.3594 MPa (green); (**B**) Two-day treated scaffold modulus is 0.0310 MPa (blue); (**C**) Seven-day treated scaffold modulus has a value of 0.0107 MPa (yellow); (**D**) Comparison of the Young’s modulus of the experiments. The number of samples is the same as the ones used in the mechanical analysis.

The Young’s modulus was obtained from the slope of the linear part of strain-stress curves, with the results shown in Figure 6 for PBS-treated scaffolds as compared to the control ones.

**Figure 7:**
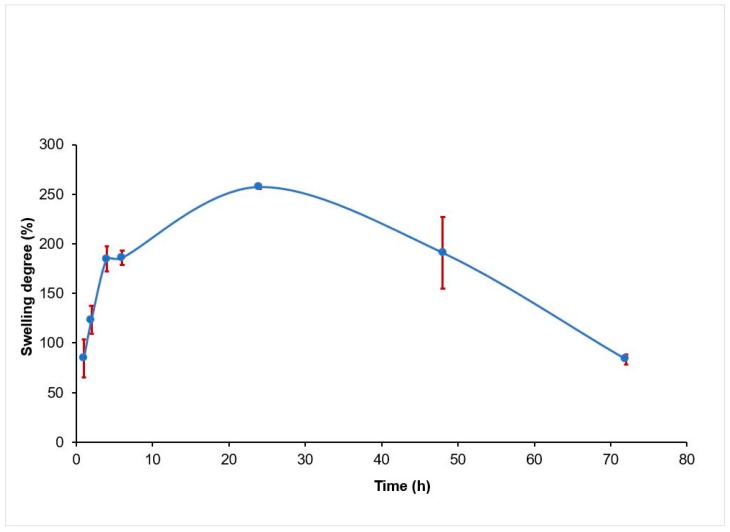
Swelling degree variation over 72 h of incubation in PBS at 37 °C, 5% CO_2_ for 2% alginate scaffolds, crosslinked in 50 mM CaCl_2_.

The swelling degree for the 2% alginate scaffolds was examined over 72 h, and shown in Figure 7, with an average of three samples. It is seen that the swelling degree increases, up to 257.16% at the first 24 h, and then decreases, down to 83% at end of 72 h.

## 4. Discussion

Scaffold design has a high influence on cell development. An interconnected pore structure and high porosity ensures cellular penetration and diffusion of nutrients to cells [5]. In physiological conditions, ionically cross-linked alginate can be dissolved by the release of the divalent ions cross-linking [24]. When cross-linked alginate is exposed to saline buffers, the exchange between sodium and calcium ions leads to the degradation of the hydrogel, as the high concentration of sodium ions in saline buffers displaces calcium ions [30]. PBS, is a balanced salt solution used to maintain pH and osmotic balance. The treatment provided by the incubation of PBS, caused the porosity of the treated scaffolds, which can be seen in Figure 2. The augmented porosity shown in the treated scaffolds (Figure 2C–H) can be clearly contrasted to the smooth surface of the non-treated scaffolds (Figure 2A–D). In addition, in the non-treated scaffolds (Figure 2C,D) and two-day treated scaffolds (Figure 2G,H), a fibrous linear structure can be observed. Literature demonstrates that freeze-drying has been used for the fabrication of porous hydrogels. Thermodynamic instability is produced by rapid cooling (causing phase separation), and as the solvent is removed by sublimation, voids are left in the regions it previously occupied [31]. A study has demonstrated a highly fibrous network of porous channels (observed by SEM microscopy) in agarose hydrogel scaffolds treated with freeze drying [31]. The scaffolds showed a positive effect on cellular attachment and direction, as the cells aligned in the pores left by the solvent. Apparently, the fibrous structure that we observed through SEM imaging in 3D-printed freeze-dried alginate scaffolds agrees with the fibrous structural configuration of freeze-dried agarose hydrogel scaffolds reported by Stokols and Tuszynski [32].

With the augmented porous structure caused by PBS and freeze-drying treatments, an improvement in cell attachment and proliferation post cell seeding was expected for the treated scaffolds. According to Figure 3, which depicts the morphology of Schwann cells, and considering that mature Schwann cells should exhibit good elongation with a centrally located oval nucleus [33], on day five of the cell culture, the two-day PBS-treated scaffolds (Figure 4E) showed better cell morphology in terms of elongation than non-treated scaffolds (Figure 4B) and seven-day treatment (Figure 4H). At day seven, all scaffolds showed an increased cell density. The augmented cellular attachment and differentiation showed in the two-day PBS treatment (Figure 4D–F), could be associated with the architecture of the scaffold (augmented porosity), caused by the treatment of PBS. According to the literature, a high, interconnected porosity encourages cell ingrowth and uniform cell distribution [30]. In the SEM images obtained for the two-day treatment, a fibrous structure can be observed for the treated scaffolds, which can also be another factor that contributes to a higher cell attachment, differentiation and elongation of Schwann cells. For the PEI treatment, cells showed better attachment after the first day of cell seeding (Figure 5A,D,G) as compared to the PBS treatment alone (Figure 5A,D,G). This could be associated with the directional fiber-like configurations of the scaffold structures, as shown in SEM images) and higher porosity. Nevertheless, cell growth development was compromised, as cells remained mostly round even after seven days. This is congruent with the previous finding, in which the survival of Schwann cells decreases with higher concentrations of PEI [27].

Young’s modulus is a measure of stiffness for a scaffold, used to describe their elastic properties when they are submitted to forces of stretching or compression. Figure 6 shows the influence of PBS treatment on the scaffold modulus. The Young’s modulus reported for 2% alginate in literature is close to 0.030 MPa [34]. A higher modulus (0.3594 MPa) was obtained with the non-treated scaffolds because the samples were not exposed to PBS before mechanical test. For the treated scaffolds, the values of modulus were consistent with values reported in literature [34], indicating a decreasing effect (approximately 40%) of PBS treatment. The decreased modulus is attributed to the degradation caused by the displacement of calcium ions in cross linked alginate [30]. In tissue engineering, the mechanical properties of the biomaterials, should match that of the host tissue [35]. Studies have demonstrated, that depending on the mechanical properties, cells could either show higher differentiation and outgrowth, or cell migration [36]. Swelling behavior is another aspect of great importance, as it controls the transport of solutes into or out of the hydrogel scaffold, aiding in the supply of nutrients and oxygen into interior regions [37,38]. With this characteristic, the hydrogel can provide a biologically active environment for cell development [39]. Swelling behavior for 2% alginate is shown in Figure 7. Scaffolds reached maximum water uptake at 24 h of incubation (250%) in PBS at 37 °C. Then, they began to lose weight, which occurred when the calcium ions (trapped in the alginate when crosslinked with calcium chloride) are diffused into the medium [30]. Swelling behavior, influences chemical and physical characteristics of scaffolds, after and prior to implantation [38]. Both parameters, when controlled, can aid in the design of scaffolds, as they need to preserve its shape and mechanical stability for a prolonged time, where cells can synthesize a sufficient amount of extracellular matrix components, which can improve the regeneration process [40].

## 5. Conclusions

This study presents the effect of PBS treatment of 3D-printed alginate scaffolds on surface morphology changes of the constructs and cellular response post cell seeding. We found that the treatment of 3D-printed alginate scaffolds for 2 days in PBS could improve cell attachment and elongation compared to the control samples and 7 days of treatment. In addition, the fibrous and directional porous micro-structures observed due to the PBS treatment can promote cell development (in terms of attachment and elongation) in the printed alginate scaffolds. For the PEI treatment, our results show that the PEI coating on the scaffolds had no significant effect on cell elongation. Nonetheless, further studies could be of interest in the future to optimize the PBS treatment time, taking into consideration the mechanical and swelling behavior of alginate scaffolds.
